# FOXM1 expression is induced by the brain microenvironment and supports CRC brain metastatic adaptation

**DOI:** 10.1007/s10585-026-10397-y

**Published:** 2026-04-27

**Authors:** Fayhaa Khair, Inbal Greenberg, Sivan Fuchs, Guy Shapira, Asia Zubkov, Noam Shomron, Michal Raz, Ido Wolf, Tami Rubinek

**Affiliations:** 1 Oncology Division, Tel Aviv Sourasky University Medical Center (Ichilov), Tel Aviv, Israel; 2 Gray Faculty of Medical and Health Sciences, Tel Aviv University, Tel Aviv, Israel; 3 Institute of Pathology, Tel Aviv Sourasky University Medical Center (Ichilov), Tel Aviv, Israel

**Keywords:** Colorectal cancer, BMs, FOXM1, Metabolic adaptation, Lipid metabolism

## Abstract

Colorectal cancer (CRC) is a leading cause of cancer-related mortality in the Western world. While brain metastasis (BMs) are relatively uncommon in CRC, they represent the fourth most frequent cause of BMs overall, and their occurrence is increasingly recognized as a complication in advanced CRC, associated with poor prognosis and limited treatment options. The brain microenvironment presents unique metabolic challenges, including low oxygen levels and restricted lipid availability. In order to survive in this hostile environment, CRC cells must acquire metabolic adaptations allowing them to survive and proliferate. To identify key drivers that enable CRC cells to metastasize to and survive within the brain, we conducted a transcriptomic screen of CRC BMs and compared it to liver metastasis (LMs). FOXM1, a transcription factor critical for tumor progression, was identified among the upregulated genes, and these results were validated by immunohistochemistry. To study the role of the brain microenvironment in mediating FOXM1 expression, we examined FOXM1 expression in CRC cell lines grown in either astrocyte-conditioned media (A-CM) or hepatocyte-conditioned media (H-CM). We observed a marked upregulation of FOXM1 following exposure to A-CM. Moreover, using an intracranial CRC BMs mouse model, a significant FOXM1 overexpression was observed. In accordance with the importance of fatty acid metabolism in BMs, our study revealed a significant correlation between FOXM1 and fatty acid synthase (FASN) in the CRC BMs, in agreement with public databases. These findings suggest that FOXM1 plays a key role in CRC BMs progression and may serve as a promising therapeutic target in this challenging disease setting.

**Supplementary Information:**

The online version contains supplementary material available at 10.1007/s10585-026-10397-y.

## Introduction

Colorectal cancer (CRC) is one of the leading causes of cancer-related mortality worldwide [1, 2]. While historically rare, brain metastasis (BMs) from CRC have emerged as the fourth most common cause of BMs following lung cancer, breast cancer, and melanoma. BMs occur in up to 4% of CRC patients [3] and are associated with a dismal median overall survival of only three to six months [3]. This grim outlook underscores the urgent need for more effective therapeutic strategies to manage and treat CRC BMs [4, 5].

In order to thrive within the brain's hostile microenvironment, metastatic CRC cells must undergo highly selective adaptations, requiring them to navigate complex biological barriers to successfully colonize and proliferate in the brain [6, 7]. These include changes in gene expression and metabolic rewiring, enabling them to overcome the unique challenges posed by the brain. Understanding the molecular mechanisms driving these processes is critical, as it could expose unique vulnerabilities that could be exploited to disrupt tumor progression. Despite significant advancements in understanding CRC progression, BMs remain an under-explored aspect, especially in comparison to the well-characterized primary tumors and liver metastasis (LMs) [8]. While the transcriptomic landscapes of primary CRC and LMs have been extensively studied, revealing key mutations including KRAS, BRAF, TP53, PIK3CA, APC, and SMAD4, the molecular mechanisms driving CRC BMs remain poorly understood [8, 9].

A recent work in our lab has shown that there is a higher IRS2 gene amplification in CRC BMs compared to other metastatic sites or to the primary tumor. This was observed based on the analysis of a genomic database of over 35,000 CRC biopsies obtained from both local and metastatic sites. Given this observed gene amplification in CRC BMs, we hypothesized that specific genes and pathways at the mRNA level promote the development of CRC BMs and that identifying these factors may reveal mechanisms mediating tropism to the brain. To this aim, we analyzed transcriptomic data from CRC BMs and LMs clinical samples and discovered upregulation of several genes, including FOXM1, in BMs compared to LMs. FOXM1, a key transcription factor in the forkhead family, plays a critical role in cell cycle progression, particularly in the transition from the G1 to S phase and during mitosis. It is frequently overexpressed in various cancers, including CRC [10], and is associated with tumor progression, metastasis, and resistance to therapy [11, 12]. In CRC, FOXM1 is associated with aggressive tumor features and metastasis, through its involvement in epithelial-mesenchymal transition (EMT), PI3K-AKT signaling, and metabolic reprogramming [10, 13]. In order to adapt to the brain’s lipid-scarce microenvironment, where extracellular lipids are limited, cancer cells undergo metabolic reprogramming, leading to increased de novo fatty acid synthesis to sustain survival and proliferation. A key enzyme that catalyzes de novo synthesis of long-chain saturated fatty acids, including palmitate from acetyl-CoA and malonyl-CoA, is fatty acid synthase (FASN), which has been shown to be regulated by FOXM1 in CRC [14].

Using in vitro and in vivo models, we demonstrated that FOXM1 expression is upregulated in CRC cells cultured in astrocyte-conditioned media (A-CM) and in intracranial mouse models of CRC BMs. This upregulation was mediated by high levels of hepatocyte growth factor (HGF) present in A-CM. Additionally, we identified a strong association between FOXM1 and FASN. Taken together, our findings underscore a critical role for FOXM1 in facilitating the brain tropism of CRC cells, positioning it as a potential therapeutic target for combating CRC BMs.

## Results

### Transcriptomic analysis of CRC BMs compared to LMs

**Fig. 1:**
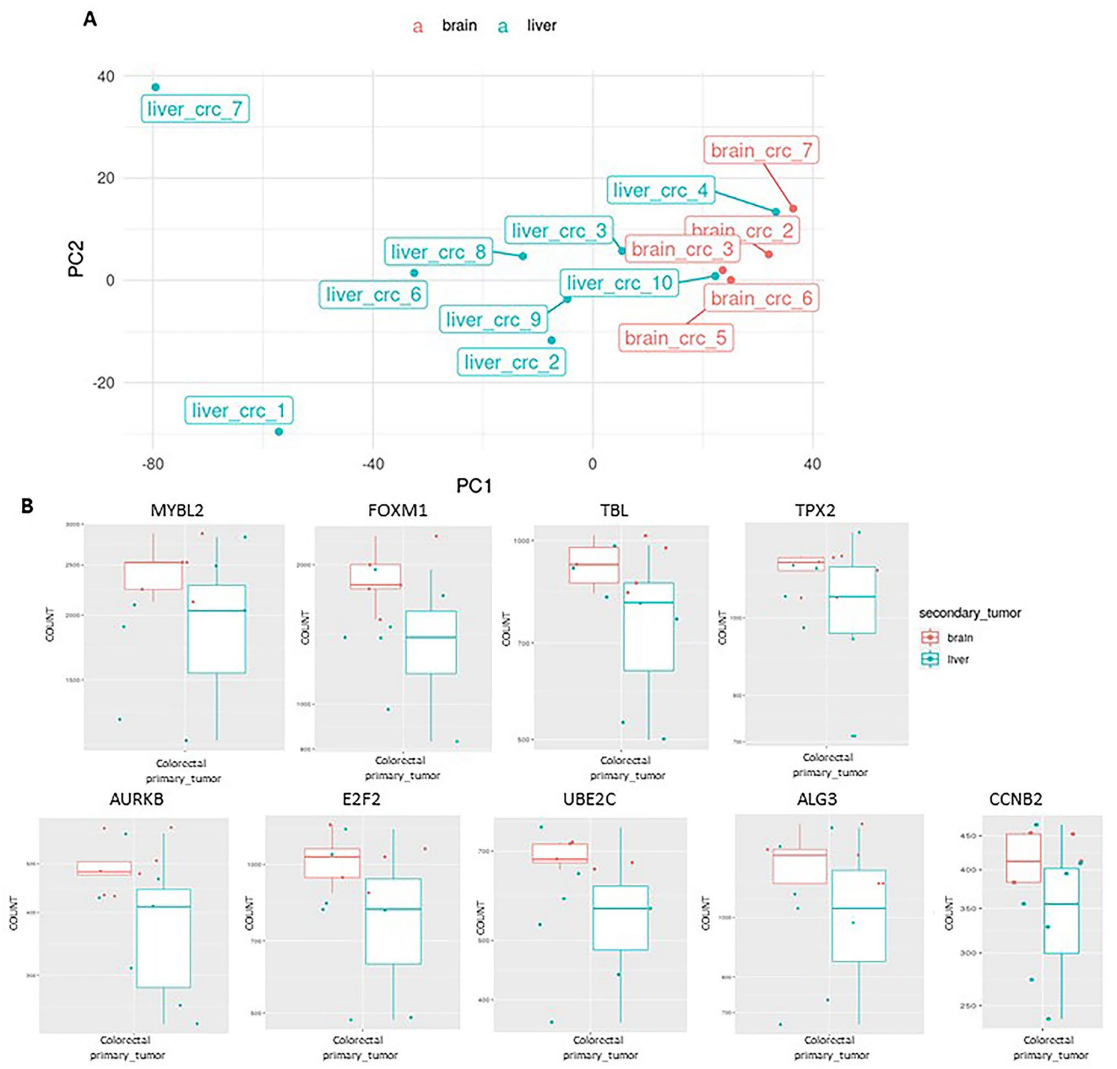
Transcriptomic profiling reveals distinct gene expression signatures in CRC brain versus liver metastasis. **A** RNA was extracted from FFPE samples of human CRC brain metastasis (BMs; n = 7) and liver metastasis (LMs; n = 10), and subjected to RNA-seq analysis. The PCA plot depicts transcriptomic variation between BMs (red) and LMs (blue), demonstrating distinct clustering by metastatic site, indicating differential gene expression profiles associated with each metastatic site. **B** Differential expression of selected genes in CRC BMs versus LMs. Box plots showing RNA-seq expression levels of nine genes (MYBL2, FOXM1, TBL1, TPX2, AURKB, E2F2, UBE2C, ALG3, and CCNB2)

To screen for potential drivers mediating CRC tropism to the brain, we performed RNA sequencing (RNA-seq) on formalin-fixed paraffin-embedded (FFPE) clinical samples of CRC BMs (n = 7) and LMs (n = 10). CRC is a highly heterogeneous disease composed of several subtypes, each driven by different molecular mechanisms or genomic alterations. To minimize heterogeneity, we focused the analysis only on tumors harboring KRAS-activating mutations (Supplementary Table S1). Principal Component Analysis (PCA) demonstrated distinct transcriptomic profiles, with BMs and LMs samples clustering separately (Fig. 1A). Sequencing quality assessment confirmed high read counts, consistent read length distributions, and efficient alignment to the reference genome, ensuring data reliability for downstream analysis (Supplementary Fig. S1). Comparing our results with reference gene expression data from the GTEx project, we ruled out some of the genes as likely contamination from the surrounding brain tissue, unrelated to the tumor itself. To completely avoid this type of contamination, any gene with the same differential expression trend as the brain vs. liver comparison was excluded. The RNA-seq data revealed differential expression of nine genes, including the transcription factor FOXM1, which was significantly upregulated in BMs compared to LMs. This finding is consistent with FOXM1's established role as an oncogene [15].

### FOXM1 is highly expressed in CRC BMs compared to LMs samples

**Fig. 2:**
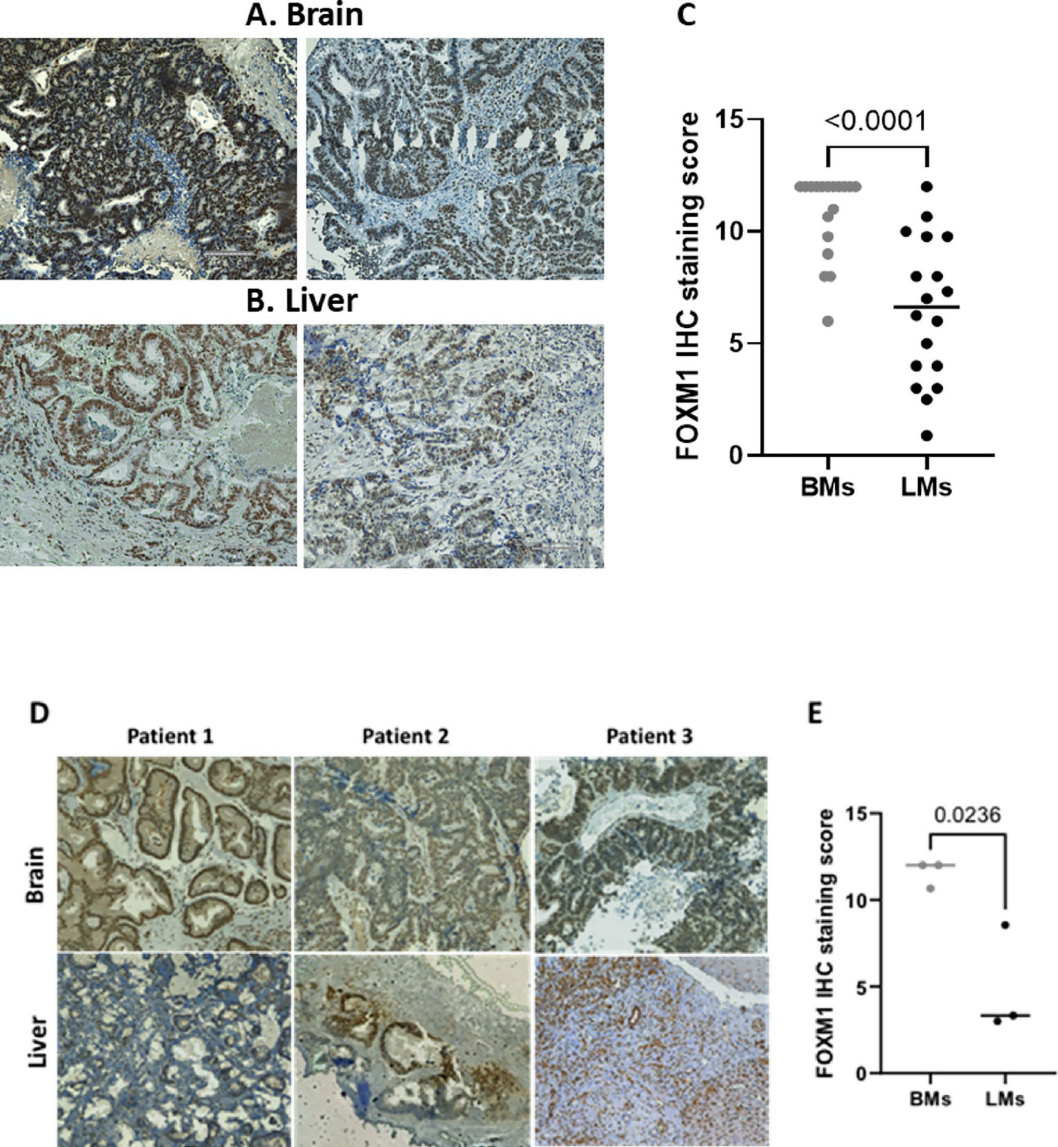
FOXM1 protein expression is elevated in CRC brain metastasis compared to liver metastasis. FFPE CRC BMs and LMs specimens (18 samples each) were immunostained for FOXM1, as described in the Methods section. Representative images of FOXM1 IHC staining in **A** BMs and **B** LMs are shown.** C** quantification of FOXM1 staining intensity was performed using IHC score. Results are displayed as mean ± s.d. (****p < *0.0001,* unpaired t-test). **D** Representative images of FOXM1 matched IHC staining in BMs and LMs from the same patient. **E** quantification of FOXM1 staining intensity was performed using IHC score. Results are displayed as mean ± s.d. (*p < *0.05,* unpaired t-test)

As the transcriptomic analysis revealed an increase in FOXM1 in BMs compared to LMs, we aimed to explore its protein expression in BMs and LMs CRC clinical samples (Supplementary Table S2, Fig. S2). To this end, we performed immunohistochemistry (IHC) on BMs and LMs samples (n = 18 each). In BMs samples, FOXM1 showed strong nuclear staining, with intensity ranging from moderate to strong in approximately 60-80% of tumor cells. In contrast, LMs samples displayed weaker staining, ranging from weak to moderate, with FOXM1 positivity observed in only 40-70% of tumor cells. (Fig. 2A-C, p < 0.0001). Additionally, we analyzed FOXM1 staining in three matched sample pairs from patients with both LMs and BMs. In each case, FOXM1 expression was higher in BMs compared to LMs (Fig. 2D,E, p < 0.05). These results confirm at the protein level what was observed at the mRNA level, reinforcing FOXM1 as a key factor associated with CRC adaptation to the brain microenvironment.

### Brain microenvironment induces FOXM1 upregulation in CRC cells *in vivo*

**Fig. 3:**
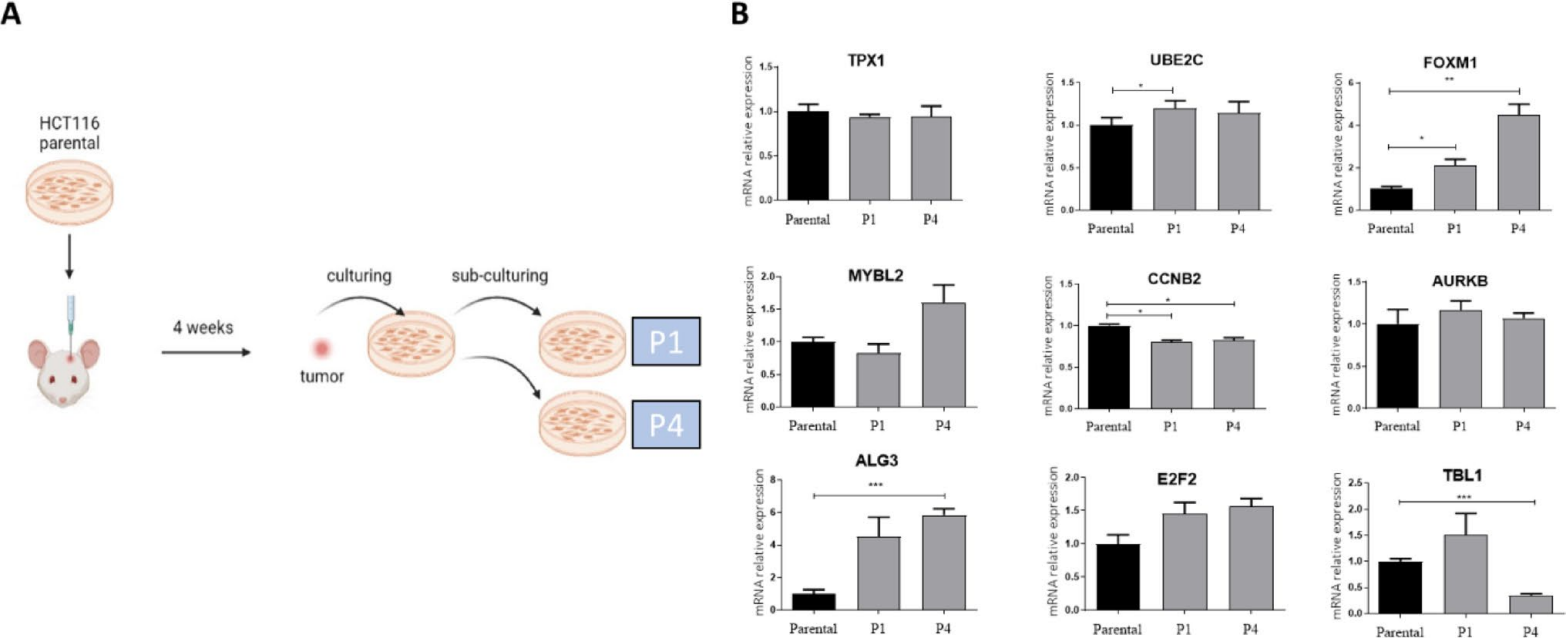
Upregulation of CRC-associated genes following adaptation to the brain microenvironment. **A** HCT116 parental cells were intracranially injected into the brains of mice (n = 3). After four weeks, tumors were excised, pooled, and tumor-derived cells were cultured and passaged up to the fourth generation.** B** RNA was extracted from first (P1) and fourth (P4) passage cells, and mRNA levels of FOXM1 and other upregulated genes were assessed by RT-PCR and normalized to β-Actin. Results are shown as fold change relative to parental cells. Experiments were repeated independently three times using cultures grown in parallel. Data are presented as mean ± s.d. (*p < 0.05, **p < *0.01*, ***p < *0.005*, unpaired t-test)

To determine whether the elevated FOXM1 levels in CRC BMs represent a primary event (i.e., cells with high FOXM1 expression preferentially metastasize to the brain) or a secondary event (i.e., the brain environment induces FOXM1 expression), we intracranially injected the CRC cell line HCT116 into the brains of mice and subsequently examined FOXM1 expression. Tumors were excised after four weeks, cultured, and analyzed at the first and fourth passages (Fig. 3A) for the expression of the nine genes identified as upregulated through RNA-seq screening (shown in Fig. 1). The results showed a significant upregulation of FOXM1 in brain-derived HCT116 cells, with a 2.0-fold increase observed at the first passage and a pronounced 4.5-fold increase by the fourth passage (Fig. 3B). These findings suggest that exposure to the brain microenvironment strongly induces FOXM1 expression. Importantly, additional three of the nine identified genes were also upregulated in the mouse brain microenvironment. MYBL2 showed a 1.7-fold increase after the fourth passage; ALG3 increased significantly by 4.8-fold after the first passage and by 6.0-fold by the fourth; and TBL1 exhibited a 1.5-fold increase after the first passage (Fig. 3B), further validating the robustness of the RNA-seq screen.

### BMs-associated genes are upregulated in astrocyte-conditioned media (A-CM) compared to hepatocyte CM (H-CM)

**Fig. 4:**
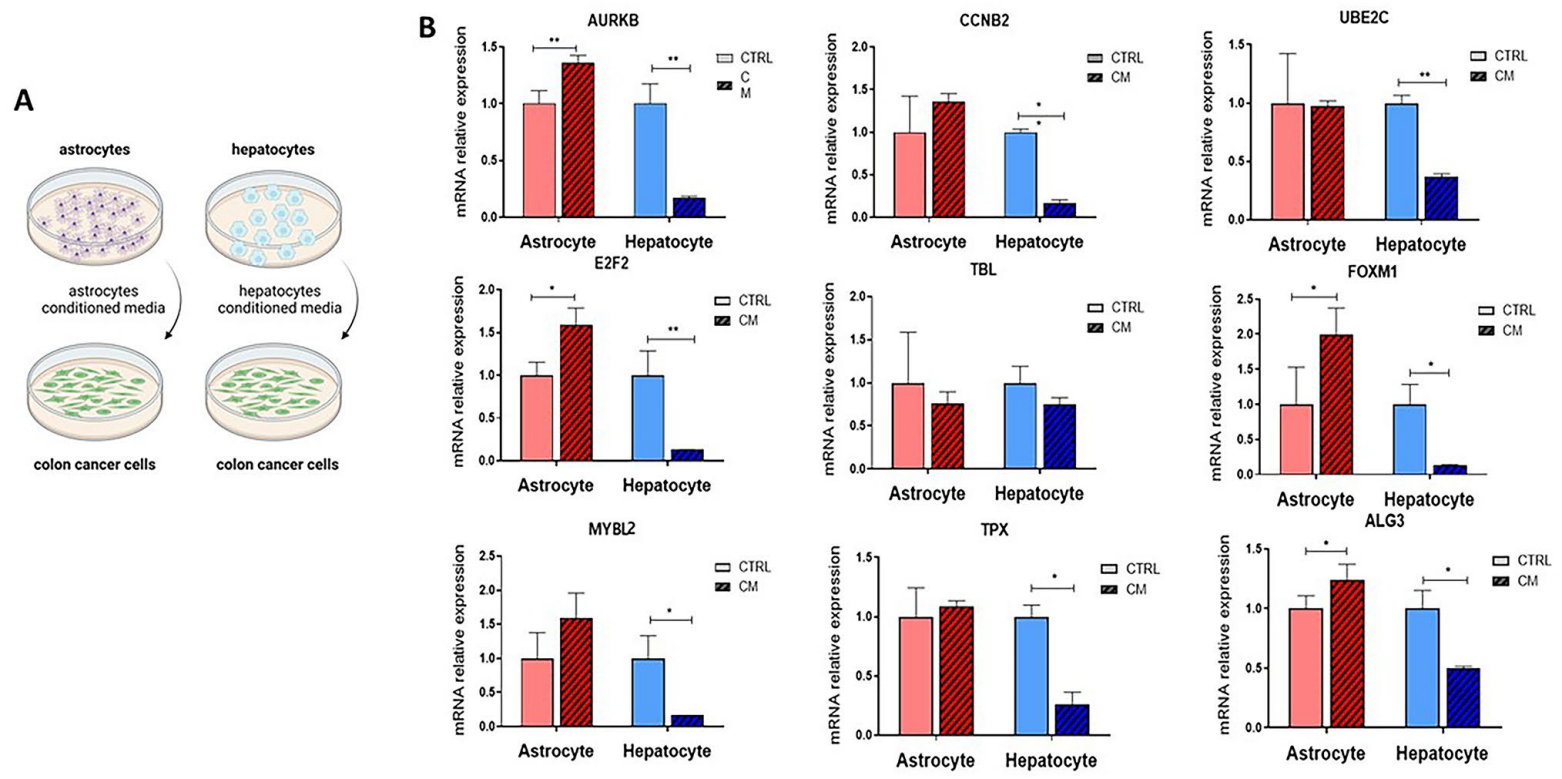
Brain microenvironment induces FOXM1 and other BMs-related genes overexpression. **A** Conditioned media (CM) were generated from astrocytes (A-CM) and hepatocytes (H-CM). Cells were cultured under standard conditions until ~ 90% confluency, after which the medium was replaced with serum-free medium (SFM). Conditioned media were collected after 24 h.** B** HCT116 cells were incubated with either A-CM or H-CM for 48 h. RNA was then extracted, and mRNA levels of FOXM1 and other CRC-related genes were measured by RT-PCR and normalized to β-actin. Results are presented as fold change relative to cells cultured in standard (control) medium. Data are shown as mean ± s.d. from at least three independent experiments (*p* < 0.05, *p* < 0.01, *p* < 0.001)

We aimed to reveal whether the upregulation of these genes was driven by soluble factors. In order to study this, HCT116 cells were grown in either astrocyte-conditioned media (A-CM) or hepatocyte-conditioned media (H-CM), and gene expression was examined (Fig. 4A). Cells cultured in A-CM exhibited significant upregulation of FOXM1, AURKB, E2F2, and ALG3 mRNA expression, whereas those cultured in H-CM showed a reduction in the expression of these genes (Fig. 4B). These findings support the role of soluble factors in the brain microenvironment in mediating the expression of this unique set of genes.

### Hypoxia induces FOXM1 expression and transcriptional activity in CRC cells

**Fig. 5:**
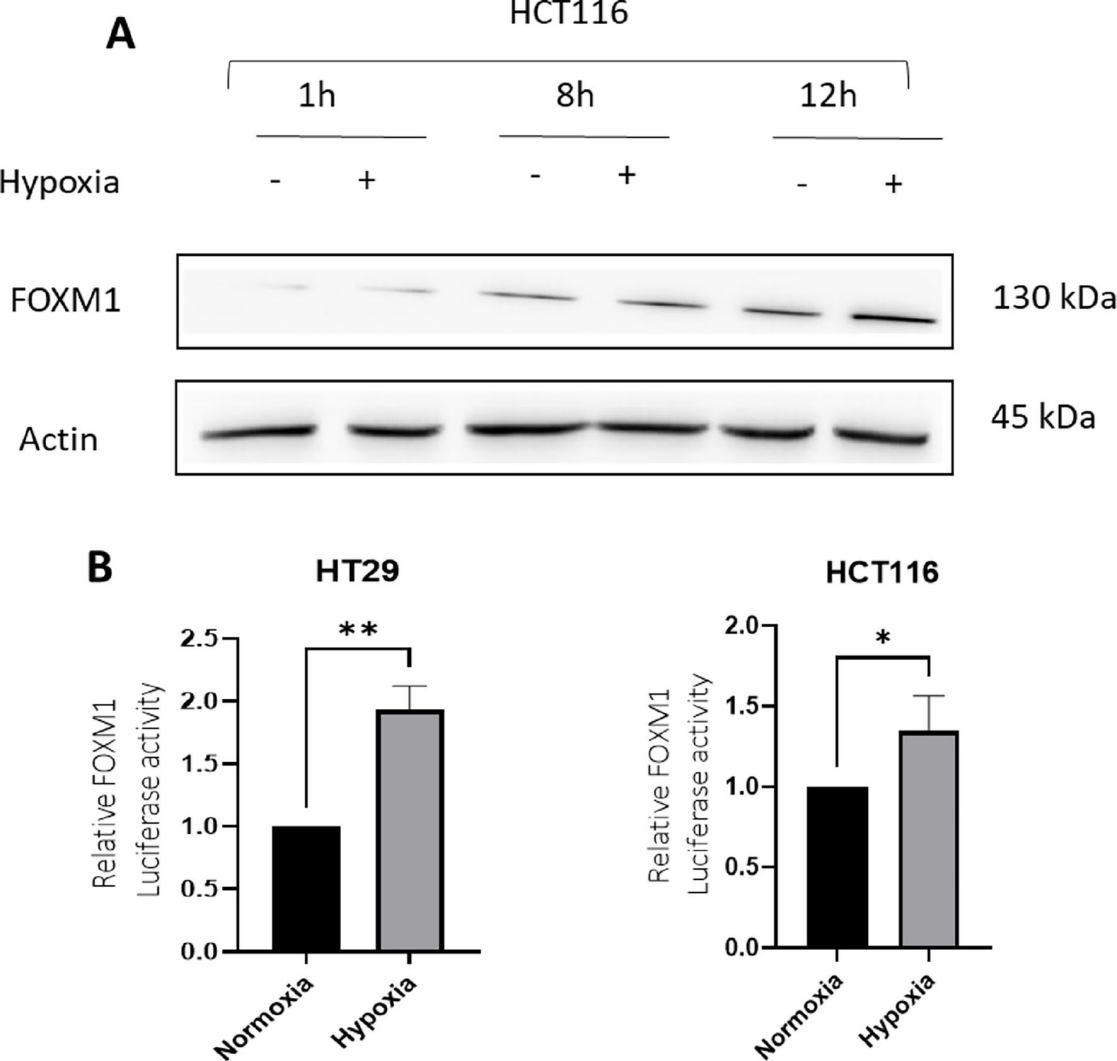
Hypoxia induces FOXM1 expression and transcriptional activity in CRC cells. HCT116 cells were seeded in a 6-well plate and incubated under normoxic or hypoxic conditions (1% oxygen) for 1, 8, or 12 h. **A** FOXM1 protein expression was assessed by WB, with β-actin used as a loading control. **B** FOXM1 transcriptional activity was measured using a luciferase reporter assay. HCT116 and HT29 cells were transfected with a plasmid containing the FOXM1 promoter upstream of a luciferase reporter gene and then exposed to normoxic or hypoxic conditions for 24 h. Luciferase activity was normalized to total protein concentration. Data are shown as mean ± s.d. (*p < 0.05, **p < *0.01*)

Our next goal was to decipher specific factors within the brain environment mediating expression of genes that promote metastasis growth in brain conditions. We focused on FOXM1, as this gene was consistently induced in brain conditions, increased in BMs clinical samples (Fig. 2) and reported to increase CRC metastasis in previous studies [10, 13]. As the brain microenvironment is characterized by low oxygen pressure, we investigated FOXM1 expression following culture of HCT116 cells under hypoxic conditions (1% O2). FOXM1 protein expression was increased in a time-dependent manner, with maximal expression observed at 12 h (Fig. 5A). We next assessed the effect of hypoxia on FOXM1 transcriptional activity using a luciferase reporter assay. Under hypoxic conditions, luciferase activity was increased twofold in HT-29 cells and 1.4-fold in HCT116 cells (Fig. 5B).

### Hepatocytes growth factor (HGF) induces FOXM1 expression and transcriptional activity in CRC cells

**Fig. 6:**
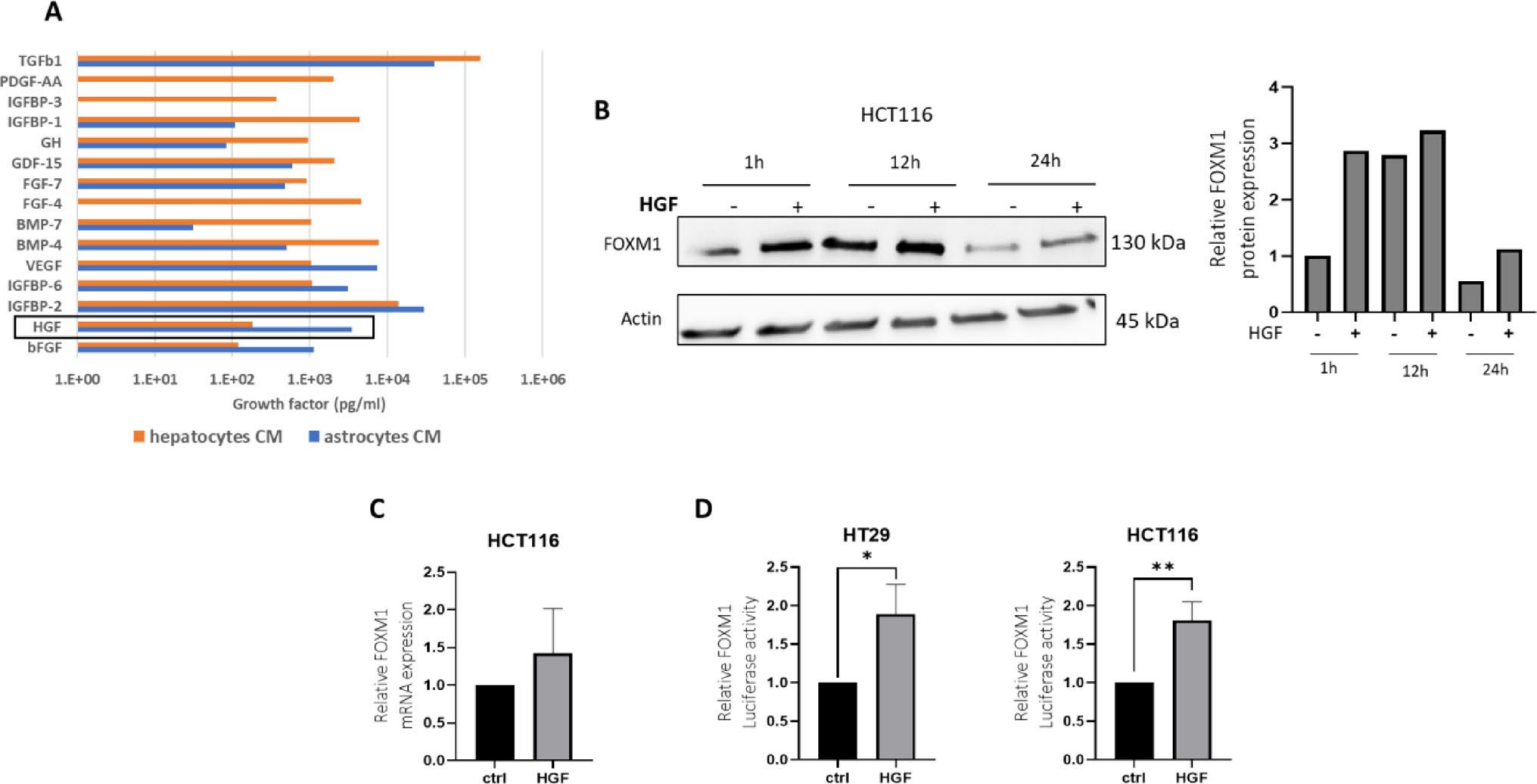
HGF upregulates FOXM1 expression and transcriptional activity in CRC cells. **A** Growth factor profile of conditioned media from astrocytes and hepatocytes. Bar chart representing the concentration (log scale) of selected growth factors measured in conditioned media from human astrocytes (blue) and hepatocytes (orange) using a growth factor array. Notably, TGFβ1, PDGF-AA, and IGFBP1 and 3 were elevated in hepatocyte-conditioned media, while bFGF and HGF showed higher levels in astrocyte-conditioned media. **B** HCT116 cells were serum-starved for 48 h, then treated with (HGF (80 ng/ml)) for 1 h, 12 h and 24 h and FOXM1 expression was studied using Western blot. Quantitation of FOXM1 protein levels were calculated relative to β-actin using ImageJ software. **C** Cells were treated as in (B) and FOXM1 mRNA levels were determined using RT-PCR. **D** HCT116 and HT29 cells were transfected with a luciferase reporter plasmid containing the FOXM1 promoter. The cells were starved for 48 h followed by HGF treatment (80 ng/ml) for 24 h. Luciferase activity was analyzed and normalized to total protein concentration. Data are shown as mean ± s.d. (*p < 0.05, **p < *0.01*).

The brain microenvironment differs from the liver microenvironment in its array of growth factors. Since FOXM1 expression was elevated in A-CM but not in H-CM (Fig. 4B), we performed a growth factors array to identify factors in A-CM that might induce FOXM1 upregulation. The analysis revealed differences in the levels of several growth factors, including increased expression of PDGF, IGFBP1, and IGFBP3 in H-CM. On the other hand, higher levels of bFGF and HGF were observed in A-CM. Thus, HGF levels in A-CM were 3489.8 pg/ml whereas in H-CM they were 182.7 pg/ml (Fig. 6A). This prompted us to study the effect of HGF on FOXM1 expression. The results showed that HGF treatment resulted in a time-dependent increase in FOXM1 protein expression in HCT116 cells, with a significant 2.8-fold upregulation observed after 1 h, which was sustained for 24 h (Fig. 6B). A similar trend was observed at the mRNA level (Fig. 6C). Additionally, HGF increased FOXM1 transcriptional activity by 1.8-fold in HT29 cells and by 1.7-fold in HCT116 cells (Fig. 6D).

### Synergistic regulation of FOXM1 by HGF and hypoxia in CRC cells

**Fig. 7:**
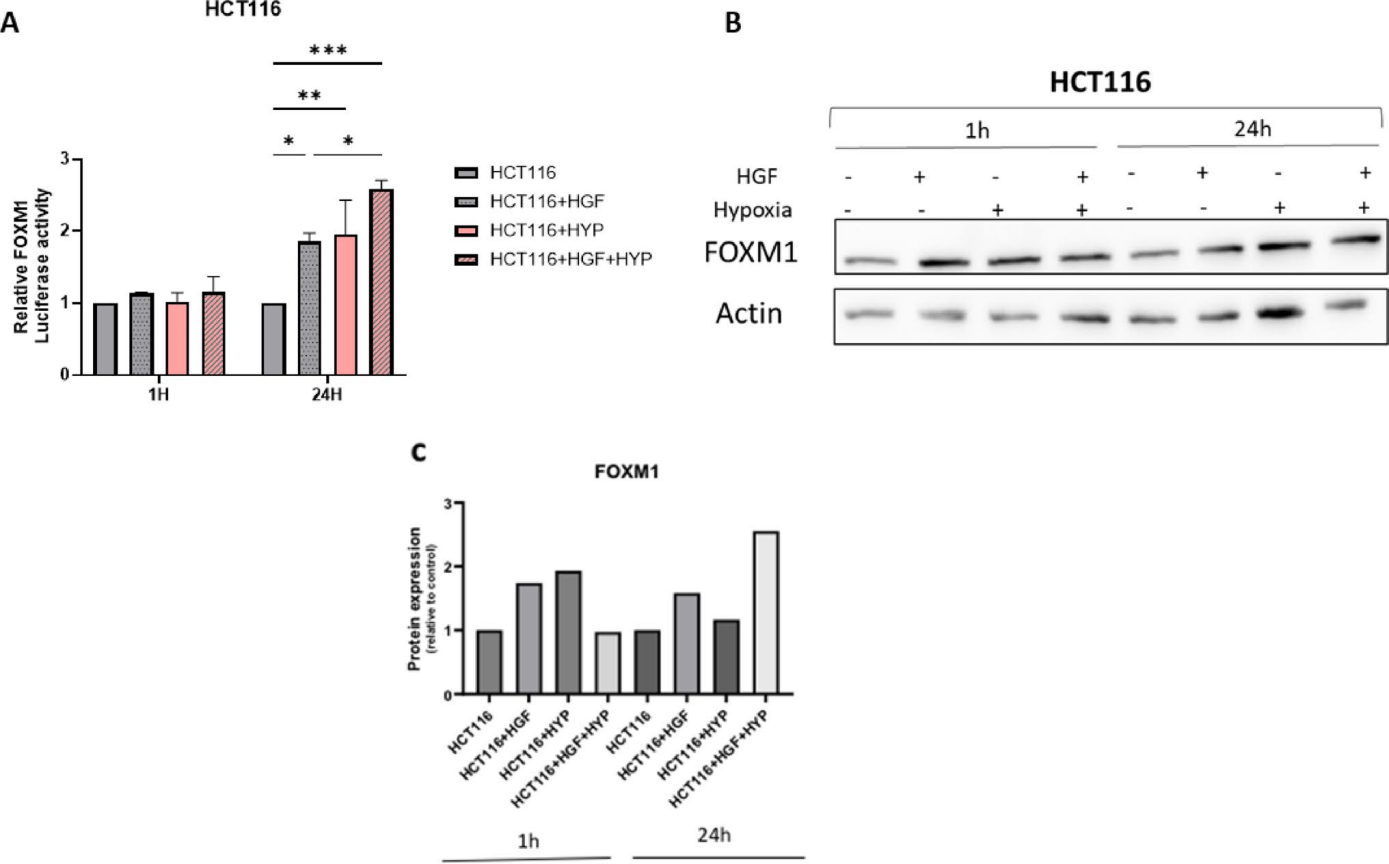
Combined HGF and hypoxia treatment cooperatively enhances FOXM1 promoter activity and protein expression in CRC cells. HCT116 cells were transfected with a plasmid containing the FOXM1 promoter upstream of a luciferase reporter gene and serum-starved for 48 h. The cells were then treated with HGF (80 nM), hypoxia (1% O₂), or both stimuli for 1 and 24 h. **A** FOXM1 promoter activity was measured using a luciferase reporter assay and normalized to total protein concentration. Both HGF and hypoxia independently increased FOXM1 transcriptional activity, with the combination producing a further additive elevation. Data are shown as mean ± s.d. (*p < 0.05, **p < 0.01, ***p < *0.005*). **B** FOXM1 protein levels were analyzed by Western blot following the same treatments. β-actin was used as a loading control. **C** Quantitation of FOXM1 protein levels relative to β-actin was calculated using ImageJ software, showing the highest expression under combined HGF + hypoxia conditions

Having established that hypoxia and HGF alone induce FOXM1 expression and transcriptional activity in CRC cells (Fig. 5 and 6), we next sought to determine whether these two key features of the brain metastatic microenvironment cooperate to regulate FOXM1 expression. To this end, HCT116 cells, transfected with a FOXM1 promoter-luciferase construct, were exposed to HGF, hypoxia (1% O₂), or their combination. While each stimulus independently increased FOXM1 transcriptional activity, combined treatment resulted in a significantly greater induction after 24 h, indicating an additive enhancement of FOXM1 promoter activation (Fig. 7A). Consistent with these transcriptional changes, Western blot analysis revealed that FOXM1 protein levels were elevated following either HGF or hypoxia alone, with the highest expression observed upon combined treatment (Fig. 7B). Quantification of FOXM1 protein levels further confirmed that concurrent exposure to HGF and hypoxia produced maximal protein induction compared to either condition alone (Fig. 7C). Collectively, these findings demonstrate that growth factor signaling and hypoxic stress converge to cooperatively amplify FOXM1 expression in CRC cells, supporting a model in which FOXM1 responds to multiple cues present in the brain metastatic microenvironment.

### FOXM1 levels correlate with FASN levels in CRC BM

**Fig. 8:**
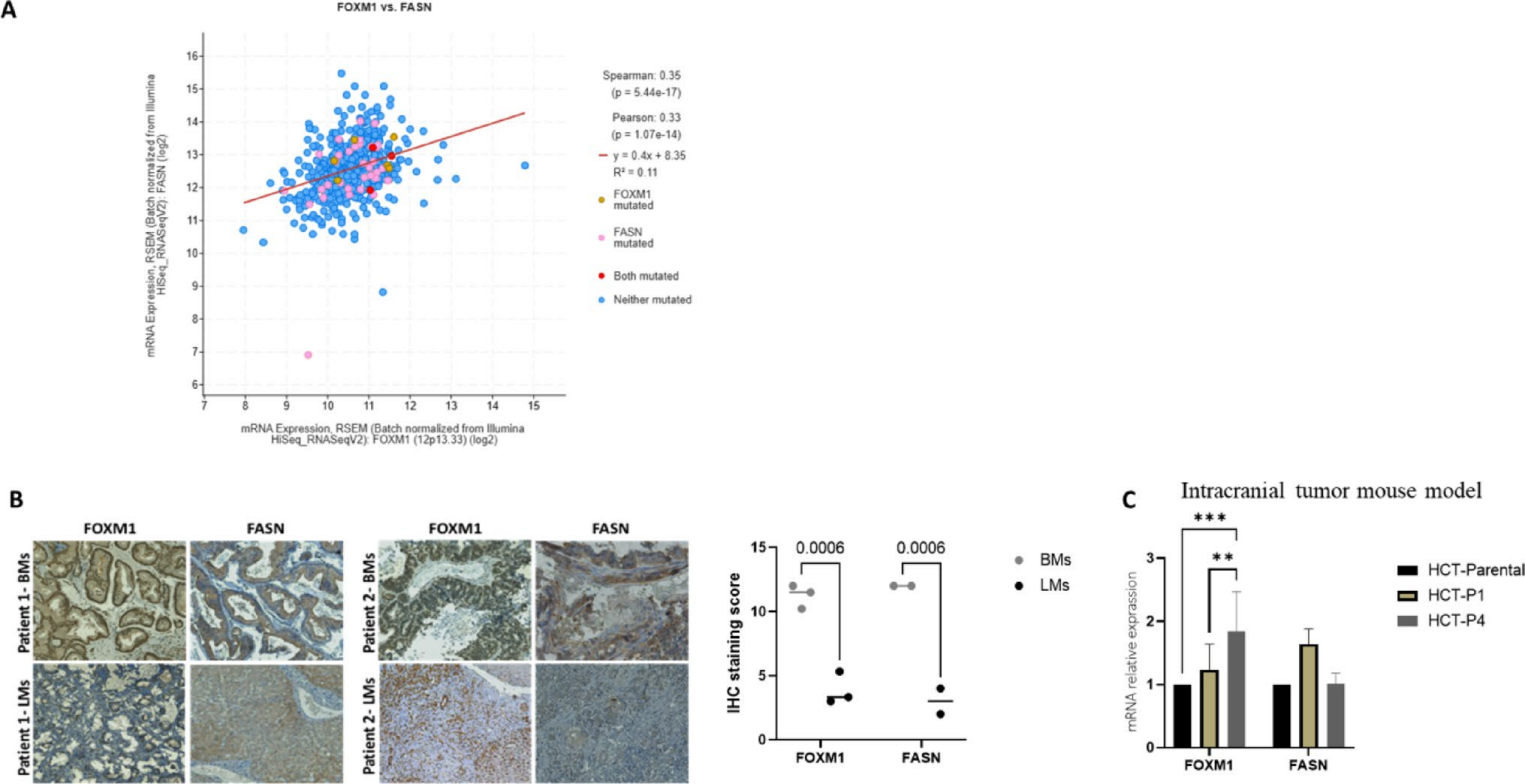
FOXM1 and FASN are co-expressed and upregulated in CRC brain metastasis. **A** Correlation between FOXM1 and FASN mRNA expression in colorectal cancer (TCGA). Scatter plot showing the relationship between FOXM1 and FASN expression (log2 RSEM values) in CRC samples from TCGA (Illumina HiSeq RNA-seq). Each dot represents an individual tumor sample. Expression of FOXM1 is plotted on the x-axis and FASN on the y-axis. Samples are color-coded based on mutation status: blue for neither gene mutated, yellow for FOXM1 mutated, pink for FASN mutated, and red for both mutated. A positive correlation is observed (Pearson r = 0.33, *p* = 1.07e- 14; Spearman ρ = 0.35, *p* = 5.44e -17). The red regression line represents the linear fit (y = 0.4x + 8.35, R^2^ = 0.11), indicating a modest but significant association between FOXM1 and FASN expression. **B** IHC staining of FOXM1 and FASN in BMs and LMs from the same patient. Quantification of FOXM1 and FASN staining intensity was performed using IHC score. Data were analyzed using two-way ANOVA and are presented as mean ± s.d. (***p < *0.001*). **C** FOXM1 and FASN expression were measured by RT-PCR before and after intracranial injection of HCT116 cells into mice brains (as shown in Fig. 3. Data were analyzed using two-way ANOVA and are presented as mean ± s.d. (*p < 0.05, **p < *0.01*)

Formation of breast cancer BMs requires de novo fatty acid synthesis, mediated through fatty acid synthase (FASN) [16], and accumulating data indicate a role for FOXM1 in regulating FASN expression in primary brain tumors [14]. We hypothesized that FOXM1 upregulates FASN expression in CRC BMs and tested this using a bioinformatic analysis of the TCGA CRC database. The analysis revealed a positive correlation, r = 0.35, between FOXM1 and FASN (p = 5.44e-17), indicating a link between FOXM1 activity and lipid metabolism in CRC (Fig. 8A). We then examined whether this correlation was observed at the protein level by comparing FOXM1 and FASN expression in matched CRC BMs and LMs specimens from the same patient. The results showed increased expression of FOXM1 and FASN in BMs, whereas their expression in LMs was decreased (Fig. 8B). Lastly, we aimed to determine whether a similar correlation was observed in the intracranial HCT116 cultured tumors (see Fig. 3). Indeed, a 1.8-fold increase in FASN expression was observed in the 1st passage, in correlation with FOXM1 expression. However, this correlation was not maintained at the 4th passage (Fig. 8C). This suggests a positive correlation between FOXM1 and FASN in CRC cells exposed to the brain environment; however, tumor cell selection during passage may favor subpopulations with lower FASN expression, possibly due to alternative metabolic adaptations or FOXM1-driven feedback mechanisms that suppress FASN over time.

## Discussion

CRC BMs represent a rare but increasingly recognized complication in advanced CRC cases, accounting for approximately 8% of all diagnosed BMs. This increase is attributed to advancements in metastatic CRC treatments, which have improved patient survival rates [17–20]. Understanding the unique characteristics of CRC BMs is crucial for developing targeted therapies. Our study provides a comprehensive analysis of the transcriptional landscape of CRC BMs, focusing on the role of FOXM1, a key transcription factor implicated in tumor progression and adaptation to the brain microenvironment [21, 22]. In this study, we demonstrate that FOXM1 is markedly upregulated in CRC BMs compared to LMs, suggesting that FOXM1 may facilitate CRC cell survival, proliferation, and adaptation to the unique brain microenvironment, which is characterized by low oxygen levels and limited nutrients [23]. This finding aligns with previous research that has identified FOXM1 as a crucial transcription factor involved in the regulation of oncogenic signaling pathways in brain tumors. Moreover, FOXM1 overexpression has been documented in breast cancer BMs, further supporting its significance in the context of brain-specific tumor progression [21]. Importantly, elevated FOXM1 expression has also been associated with poor prognosis in colorectal cancer patients in general, further underscoring the potential clinical relevance of FOXM1 upregulation in CRC brain metastasis (Koo et al., BBA, 2012; Nandi et al., Semin Cancer Biol., 2018).

In this study, LMs were used as a biologically relevant reference site to BMs, as the liver represents the most common and permissive metastatic niche for colorectal cancer, facilitated by direct portal circulation and a metabolically supportive microenvironment, whereas the brain constitutes a distant and highly restrictive site characterized by hypoxia, limited lipid availability, and blood-brain barrier protection. Comparing BMs to LMs therefore allowed us to distinguish general metastatic features from brain-specific adaptive programs. Although comparison with matched primary tumors would provide additional insight into early metastatic events, such samples are rarely available for patients with CRC BMs, which typically arise at advanced or terminal disease stages. Importantly, previous analysis of TCGA datasets indicate that FOXM1 is already upregulated in metastatic CRC compared to primary tumors, supporting its established role in disease progression. Our findings extend these observations by demonstrating a further induction of FOXM1 specifically within the brain microenvironment, driven by local cues such as astrocyte-derived HGF and hypoxia.

Using RNA-seq, we identified FOXM1 as significantly upregulated in CRC BMs compared to LMs, along with several other genes. These findings are consistent with FOXM1’s established role as an oncogene and suggest it may play a critical role in CRC adaptation to the brain’s unique conditions, which are characterized by hypoxia and limited nutrients. To validate our RNA-seq findings, we performed IHC on clinical samples. FOXM1 protein expression was markedly elevated in CRC BMs compared to LMs, which was further supported by paired patient samples of both BMs and LMs, which similarly demonstrated increased FOXM1 expression in BMs. These results emphasize FOXM1’s potential role in mediating CRC progression and adaptation within the brain microenvironment.

To explore the mechanisms underlying FOXM1 regulation, we examined the role of brain-derived factors. CRC cells cultured in A-CM displayed significant upregulation of FOXM1 compared to cells cultured in H-CM. These results suggest that soluble factors specific to the brain microenvironment drive FOXM1 expression, enhancing CRC cell adaptation to the brain.

Hypoxia, a hallmark of the brain microenvironment, was found to be another critical regulator of FOXM1 expression. We observed a time-dependent increase in FOXM1 protein expression and transcriptional activity under hypoxic conditions, which is consistent with previous studies demonstrating that FOXM1 transcriptional activation under hypoxia is mediated by HIF-1, suggesting a potential mechanism by which CRC cells adapt to the low oxygen environment of the brain [24]. While physiological oxygen levels in the brain can vary, we used 1% O₂ as a defined hypoxic condition that reliably activates HIF-1-dependent pathways relevant to FOXM1 regulation and metastatic adaptation. Our findings suggest that the upregulation of FOXM1 in CRC BMs is not solely a consequence of selection for FOXM1-high cells but also a response to brain-specific factors such as HGF and hypoxia. Analysis of astrocyte-conditioned medium (A-CM) in our system demonstrated elevated levels of HGF, along with other growth factors such as bFGF, indicating that multiple astrocyte-secreted factors may influence FOXM1 regulation. We focused on HGF because its stimulation directly induced FOXM1 expression in CRC cells, further highlighting the influence of the brain microenvironment on FOXM1-mediated processes and suggesting a specific interaction between CRC cells and brain-derived factors that enhances FOXM1 expression and activity. This observation aligns with findings in pancreatic cancer, where Huang et al. demonstrated a positive feedback loop between HGF/Met signaling and FOXM1, leading to increased cancer cell aggressiveness and resistance to Met inhibition [25]. This suggests that similar mechanisms may be at play in CRC BMs, potentially contributing to their adaptation and survival in the brain microenvironment.

To directly investigate whether FOXM1 is overexpressed in CRC cells within the brain microenvironment, we used an intracranial CRC BMs mouse model followed by RT-PCR to evaluate FOXM1 levels before and after injection. Although intracranial injection bypasses critical early metastatic steps- such as acquisition of metastatic traits, hematogenous dissemination, and blood-brain barrier (BBB) extravasation- it remains widely used due to the lack of spontaneous or reproducible orthotopic CRC models that reliably form brain metastasis. Additionally, direct injection into the brain may induce local inflammation, which can complicate studies focused on neuroinflammatory processes. Nevertheless, given the rarity of CRC BMs and the scarcity of experimental systems available to model them, the intracranial approach is currently the most viable and reproducible strategy for studying tumor adaptation and growth within the brain. This model enables controlled and consistent establishment of lesions, thereby allowing mechanistic interrogation of tumor-brain microenvironment interactions and evaluation of therapeutic responses in established metastasis-goals that cannot be achieved using existing vascular dissemination or orthotopic models. Consistent with this, FOXM1 expression was elevated in CRC cells following intracranial injection and tumor formation, supporting a role for FOXM1 in facilitating CRC cell adaptation and survival within the brain microenvironment.

Moreover, FOXM1 appears to play a pivotal role in metabolic reprogramming necessary for CRC cells to thrive in the brain. Our study found that FOXM1 expression correlates with FASN levels, at the mRNA and protein levels, suggesting that FOXM1 may drive metabolic changes that support CRC cell growth in the brain. This aligns with previous studies indicating that increased de novo fatty acid synthesis is crucial for cancer cells metastasizing to the brain [14]. The brain’s lipid-poor environment poses a significant challenge for metastasizing cancer cells, necessitating adaptations such as increased fatty acid synthesis [16]. Hence, this FOXM1-FASN positive correlation may be a potential mechanism in CRC cells that metastasize to the brain. It is important to note that the FOXM1-FASN relationship observed in CRC BMs and in early ex vivo passages reflects a transient metabolic state driven by the brain microenvironment. Immediately after isolation from brain lesions (passage1), both FOXM1 and FASN were elevated, consistent with their co-activation in vivo under conditions of hypoxia, HGF enrichment, and limited lipid availability. However, by passage 4, when these microenvironmental cues are lost, FASN expression decreased toward baseline while FOXM1 levels remained relatively stable. This divergence suggests that FASN upregulation is highly dependent on external metabolic pressure, whereas FOXM1 retains broader functions in cell-cycle regulation, stress responses, and transcriptional plasticity that persist independently of the brain niche. Overall, these data suggest that the FOXM1-FASN association is context-dependent, maintained under brain-specific conditions but modulated during culture adaptation as selective pressures change. Although our findings demonstrate a strong correlation between FOXM1 and FASN expression in CRC BMs, it is important to note that FOXM1 does not directly bind to the FASN promoter. Li et al. (Cell Death Discovery, 2023) showed using ChIP assays that FOXM1 regulates fatty acid synthesis indirectly through the SET7-H3K4me1-FASN axis rather than by promoter binding. These results suggest that FOXM1 may enhance fatty acid synthesis by promoting epigenetic activation of FASN, providing an additional mechanistic explanation for the metabolic adaptations observed in CRC BMs.

In conclusion, our study identifies FOXM1 as a critical factor in CRC BMs development, and elucidates its role in facilitating CRC BMs through metabolic reprogramming and adaptation to the brain microenvironment, offering new avenues for therapeutic intervention.Targeting FOXM1 could potentially disrupt the metabolic adaptations necessary for CRC survival in the brain, providing a novel strategy to combat this challenging aspect of CRC progression. Further research is needed to explore the therapeutic potential of inhibiting FOXM1 and its downstream pathways in clinical settings.

## Limitation of the study

This study has several important limitations. First, CRC BMs are extremely rare, and matched brain-liver metastatic samples from the same patients are exceedingly difficult to obtain. In most cases, CRC BMs arise at very late stages of disease progression, and patients often present with only a single metastatic site available for analysis. Consequently, although the size of our clinical cohort is limited, it represents a uniquely valuable and well-characterized collection given the scarcity of matched CRC brain and liver metastasis.

Second, the intracranial mouse model used in this study does not recapitulate early steps of the metastatic cascade, including hematogenous dissemination and blood-brain barrier (BBB) extravasation. Importantly, this limitation reflects not only experimental constraints but also the clinical reality of CRC BMs. In patients, CRC BMs develop after a prolonged disease course and represent a late and infrequent event, making it currently impossible to generate spontaneous or reliable vascular dissemination models in mice. To date, no spontaneous or robust intracardiac CRC BMs models exist, and alternative approaches such as intracarotid injection are technically challenging and poorly reproducible. Under these conditions, direct intracranial injection remains the most feasible and reliable experimental strategy for studying established CRC brain lesions. While this model bypasses early metastatic steps and may induce local neuroinflammation, it closely reflects the clinical scenario of advanced BMs and is well suited to address our specific research focus on post-seeding tumor adaptation, tumor-brain microenvironment interactions, and therapeutic responses.

Finally, although RNA-seq sample numbers were limited, several measures were taken to reduce biological and technical variability, including analysis of tumors with a comparable molecular background and application of Benjamini-Hochberg correction for multiple testing. Importantly, FOXM1 upregulation was independently validated at the transcriptomic, protein, and in-vivo levels, supporting the robustness of our findings. A further limitation is that all analyzed samples harbored KRAS mutations, a restriction applied to minimize molecular heterogeneity; however, this may limit the generalizability of the results to other CRC molecular subtypes.

## Materials and methods

### Patients clinical data and tumor specimens

Tumor samples were provided in the form of formalin-fixed paraffin-embedded (FFPE) blocks after written informed consent had been obtained from the research subjects by the Tel Aviv Sourasky Medical Center, under an approved institutional review board (IRB) (0137-21-TLV). Clinical data were obtained from patients electronic medical records. For the IHC, samples were obtained from 57 patients as follows: 21 patients had CRC BMs, and another 21 suffered from CRC LMs. Samples for RNA-seq were obtained from 7 KRAS-mutant CRC BMs and 10 KRAS-mutant CRC LMs. We searched for KRAS mutation samples to reduce heterogeneity. Tissues were collected from specimens scheduled for discard after routine surgical pathology diagnosis.

### Transcriptomic analysis

BMs specimens obtained from KRAS-mutant CRC patients (n = 7) and LMs obtained from KRAS-mutant CRC patients (n = 10), were acquired from FFPE blocks. Prior to RNA extraction, FFPE blocks were reviewed by an experienced pathologist to identify and mark areas containing at least 80% tumor cells. Unstained 3.5-mm-thick FFPE sections were prepared. The areas of interest were carefully microdissected under a dissecting microscope to ensure that only the relevant tissue was included for RNA extraction. This step was crucial to minimize contamination from surrounding non-target tissue and to enrich for the specific cell populations of interest. RNA was then extracted from these microdissected FFPE tissue sections using the High Pure RNA Isolation Kit (Roche, Mannheim, Germany) according to the manufacturer’s protocol. Briefly, the microdissected tissue was deparaffinized using xylene, followed by ethanol washes. The tissue was then digested with proteinase K, and the RNA was purified using spin columns. The quantity and quality of the extracted RNA were assessed using a NanoDrop spectrophotometer and an Agilent 2100 Bioanalyzer. RNA-seq was conducted at the Tel-Aviv University Genomics Research Unit and the Bioinformatics Unit (Tel-Aviv, Israel). The libraries were prepared using NEBNext® ULTRA™ II RNA Library Prep kit for IIumina® (New England BioLabs® Inc., USA). For sequencing, briefly, 1000 ng of total RNA was fragmented, followed by reverse transcription and second strand cDNA synthesis. The double-stranded cDNA was subjected to end repair, A-base addition, adapter ligation, and PCR amplification to create barcoded libraries. Libraries were evaluated by Qubit and TapeStation. Sequencing was conducted with the NextSeq 500/550 v2.5 (Illumina, USA) at 75-cycle Single-Read kit.

Bioinformatics analysis: The output was ~ 21 million aligned reads per sample. Adapters were identified and removed from the raw sequence reads using fastp 0.19.6 [26] Trimmed reads were aligned to the human reference genome hg38 (Ensembl gene annotations) using STAR 2.7.1a [27]. [28] Pre-alignment and post-alignment quality control (QC) reports were generated using MultiQC [29]. Genes that were differentially expressed between the BMs and the LMs were characterized using the R package DESeq2 [30]. By identifying genes with |log2FC|> 1 and a P-adjusted value < 0.05. Pathway analysis of the differentially expressed genes was performed using the R package clusterProfiler [31]. Bioinformatic analysis was done in collaboration with Prof. Noam Shomron. A list of genes differentially expressed in CRC BMs compared to CRC LMs was generated. Genes were further analyzed for functionality.

### TCGA correlation analysis of FOXM1 and FASN expression

To assess the relationship between FOXM1 and FASN expression in colorectal cancer, we used publicly available RNA-seq data from The Cancer Genome Atlas (TCGA), specifically the Colorectal Adenocarcinoma (COAD + READ) cohort. Gene expression data (log2-transformed RSEM values, batch-normalized from Illumina HiSeq RNASeqV2) were retrieved via cBioPortal (https://www.cbioportal.org). Expression values for FOXM1 and FASN were plotted, and the Pearson and Spearman correlation coefficients were calculated to assess the strength and direction of the association. A linear regression line was fitted to visualize the trend, and the coefficient of determination (R^2^) was reported.

### Cell lines

Cell lines were originally obtained from the American Type Culture Collection and authenticated with the DNA markers used by ATCC (Manassas, VA, USA). The human colorectal cancer cell lines HCT116 and HT29 CRC cell lines were cultured in DMEM medium (Gibco, Thermo Fisher Scientific, USA), containing 10% fetal calf serum (FCS) (Gibco, Thermo Fisher Scientific, USA) . All cells were grown at 37°C in a humidified 5% CO2 atmosphere.

### Astrocytes and hepatocytes conditioned media

Primary human astrocytes and their media were purchased from ScienCell Research Laboratories (Carlsbad, CA, USA). Cells were cultured in astrocyte growth medium according to the manufacturer's instructions. Primary hepatocytes were purchased from Lonza Cell Culture Products (Basel, Switzerland) and were cultured following the manufacturer’s instructions. For CM preparation, cells were grown in maintenance media, and media were collected every 24 h, over three days. Media were centrifuged and the supernatant was stored in -80C for further experiments, while maintenance media were stored for control experiments.

### Growth factor array

Conditioned media (CM) from astrocytes and hepatocytes were collected to assess secreted growth factors using the RayBiotech Quantibody® Human Growth Factor Array (RayBiotech, Inc., Peachtree Corners, GA, USA), following the manufacturer’s instructions. Briefly, each sample was incubated on a pre-coated antibody array slide targeting a panel of human growth factors. After binding, biotin-conjugated detection antibodies were applied, followed by incubation with Cy3-labeled streptavidin. Fluorescent signals were detected using a compatible laser scanner, and data were analyzed using RayBiotech’s Quantibody Q-Analyzer software. Concentrations of individual growth factors were determined based on a standard curve and expressed in pg/mL. Signal intensities were normalized to internal controls and blank-subtracted prior to statistical analysis.

### Intracranial mouse model

HCT116 cells were injected (100,000 cells in 5 μL) stereotactically into the brain of athymic nude mice at a depth of 3.5 mm, over 2 min. Tumor growth was monitored using MRI every 10 days, and after 4 weeks, tumors were excised for further in vitro experiments. The institutional committee for animal use of Tel Aviv University approved the experimental procedures in mice.

### Transfections and plasmids

Transfections were performed using the polyethyleneimine (PEI, Polyscience, Inc., Warrington, PA, USA) reagent. Plasmids used: the luciferase reporter plasmid of FOXM1 promoter was generated by VectorBuilder (Isenburg, Germany). A 1038-bp fragment of the human FOXM1 promoter (− 1012 to + 26 relative to the transcription start site) was designed based on the cloning strategy described by Wierstra and Alves [24]. The promoter sequence was synthesized and cloned into the VB240114-1014vqp plasmid, using KpnI and HindIII restriction sites flanking the insert.

### Quantitative Real-Time reverse transcription-PCR (qRT-PCR):

Total RNA was extracted using the High Pure RNA Isolation Kit (Roche, Mannheim, Germany) and was processed to cDNA with the qScript cDNA Synthesis Kit (Quantabio, Beverly, MA, USA) both according to the manufacturer's instructions. qRT- PCR was used to determine mRNA levels. Primers were designed using and synthesized by IDT (Coralville, IA, USA). Amplification reactions were performed in triplicate using StepOne Plus (Applied Biosystems, Foster City, CA, USA). PCR conditions were as follows: 50°C for 2 min, 95°C for 2 min, followed by 40 cycles of 95°C for 15 s, 60°C for 45 s.

#### Western blot analysis

Cells were harvested and lysed for total protein extraction in RIPA buffer together with a protease and phosphatase inhibitor cocktail (Sigma, St. Louis, MO, USA). Fifty μg of protein extract was loaded on 10% SDS-PAGE gels, separated electrophoretically, and blotted from the gel onto a nitrocellulose membrane (BioTraceTM NT, PALL Corporation, Port Washington, NY, USA). The membranes were immunoblotted with the indicated antibodies.

#### Immunohistochemistry (IHC)

The slides were deparaffinized in xylene (Bio-Lab Ltd, Jerusalem, Israel) and rehydrated in graded concentrations of alcohol. Antigen retrieval was performed using a 10 mM sodium citrate buffer solution at (pH 6.0). Sections were placed in a 3% hydrogen peroxide for 30 min to quench any endogenous peroxidase activity, followed by several washes of PBS with Tween 20 (PBST) and then incubated with 2.5% normal horse serum (ImmPRESS universal polymer kit peroxidase, Vector Laboratories, Newark, CA, USA) for 20 min. Then, the slides were incubated with primary antibodies diluted in antibody diluent (Zytomed Systems, Berlin, Germany) overnight. Antibodies used: anti-FOXM1 antibody (sc-376471, Santa Cruz, Dallas, TX, USA, 1:250) and anti-FASN antibody. Next, slides were incubated with horseradish peroxidase (HRP) for 30 min, followed by two washes of PBS with Tween 20 (PBST) and stained with diaminobenzidine (DAB) (ImmPACT DAB Kit) and hematoxylin (Merck, Darmstadt, Germany). The samples were quantitatively analyzed using the immunoreactive score (IRS). IRS gives a range of 0-12 as a product of multiplication between the proportion score of positive cells (0-4) and the staining intensity score (0-3) [32].

#### Luciferase assay

Cells were plated in 12-well plates and transfected with a luciferase reporter plasmid of FOXM1 promoter (VectorBuilder, Isenburg, Germany). Following transfection, cells were serum-starved, and subsequently subjected to the indicated experimental conditions. Luciferase activity was measured using the Luciferase Assay System kit (Promega, Madison, WI, USA) according to the manufacturer’s instructions. Luciferase units were normalized to total protein concentration. Representative results of at least three independent experiments are presented.

#### Statistical analysis

Data were analyzed using GraphPad Prism software, using two-tailed t-tests and One Way or two Way ANOVA followed Tukey's post hoc multiple-comparisons test. Results were considered statistically significant at *P*-values * ≤ 0.05, ** ≤ 0.01, *** ≤ 0.001, **** ≤ 0.0001. Bar graphs represent the mean and standard deviation (SD) across multiple independent experimental replicates.

## Supplementary Information

Below is the link to the electronic supplementary material.

Supplementary file1 (PDF 7863 kb)

## References

[1] Li J, Chen D, Shen M (2022) Tumor microenvironment shapes colorectal cancer progression, metastasis, and treatment responses. Front Med. 10.3389/fmed.2022.869010 35402443 10.3389/fmed.2022.869010 PMC8984105

[2] Ferlay J, Ervik M, Lam F, et al. Global Cancer Observatory: Cancer Today. Lyon, France: International Agency for Research on Cancer. Available from: https://gco.iarc.fr/today, accessed [29 09 2021]. International Agency for Research on Cancer.

[3] Christensen TD, Spindler KLG, Palshof JA, Nielsen DL (2016) Systematic review: brain metastases from colorectal cancer—incidence and patient characteristics. BMC Cancer. 10.1186/s12885-016-2290-5 27037031 10.1186/s12885-016-2290-5 PMC4818396

[4] Steeg PS (2006) Tumor metastasis: mechanistic insights and clinical challenges. Nat Med. 10.1038/nm1469 16892035 10.1038/nm1469

[5] Lowery FJ, Yu D (2017) Brain metastasis: unique challenges and open opportunities. Biochimica et Biophysica Acta (BBA) - Reviews on Cancer. 10.1016/j.bbcan.2016.12.001 27939792 10.1016/j.bbcan.2016.12.001 PMC5272787

[6] Cox TR, Gartland A, Erler JT. The pre-metastatic niche: is metastasis random? *Bonekey Rep*. 2012;1(5). 10.1038/bonekey.2012.80 10.1038/bonekey.2012.80 PMC4816289 27127624

[7] Fidler IJ (2003) The pathogenesis of cancer metastasis: the “seed and soil” hypothesis revisited. Nat Rev Cancer. 10.1038/nrc1098 12778135 10.1038/nrc1098

[8] Malki A, Elruz RA, Gupta I, Allouch A, Vranic S, Al Moustafa AE. Molecular mechanisms of colon cancer progression and metastasis: Recent insights and advancements. *Int J Mol Sci*. 2021;22(1). 10.3390/ijms22010130 10.3390/ijms22010130 PMC7794761 33374459

[9] Wang R, Li J, Zhou X et al (2022) Single-cell genomic and transcriptomic landscapes of primary and metastatic colorectal cancer tumors. Genome Med. 10.1186/s13073-022-01093-z 35974387 10.1186/s13073-022-01093-z PMC9380328

[10] Koo CY, Muir KW, Lam EWF (2012) FOXM1: from cancer initiation to progression and treatment. Biochimica et Biophysica Acta (BBA) - Gene Regulatory Mechanisms. 10.1016/j.bbagrm.2011.09.004 21978825 10.1016/j.bbagrm.2011.09.004

[11] Raychaudhuri P, Park HJ (2011) FoxM1: a master regulator of tumor metastasis. Cancer Res. 10.1158/0008-5472.CAN-11-0640 21712406 10.1158/0008-5472.CAN-11-0640 PMC3129416

[12] Laoukili J, Kooistra MRH, Brás A et al (2005) FoxM1 is required for execution of the mitotic programme and chromosome stability. Nat Cell Biol. 10.1038/ncb1217 15654331 10.1038/ncb1217

[13] Zhang C, Wang Y, Feng YF et al (2016) Gli1 promotes colorectal cancer metastasis in a Foxm1-dependent manner by activating EMT and PI3k-AKT signaling. Oncotarget. 10.18632/oncotarget.13348 27863385 10.18632/oncotarget.13348 PMC5349902

[14] Li X, Su W, Wu H et al (2023) FOXM1 maintains fatty acid homoeostasis through the SET7-H3K4me1-FASN axis. Cell Death Discov. 10.1038/s41420-023-01540-9 37620304 10.1038/s41420-023-01540-9 PMC10449838

[15] Nandi D, Cheema PS, Jaiswal N, Nag A (2018) FoxM1: repurposing an oncogene as a biomarker. Semin Cancer Biol. 10.1016/j.semcancer.2017.08.009 28855104 10.1016/j.semcancer.2017.08.009

[16] Ferraro GB, Ali A, Luengo A et al (2021) Fatty acid synthesis is required for breast cancer brain metastasis. Nat Cancer. 10.1038/s43018-021-00183-y 34179825 10.1038/s43018-021-00183-y PMC8223728

[17] Li W, Wang T, Zhu Y et al (2022) Brain metastasis from colorectal cancer: treatment, survival, and prognosis. Medicine. 10.1097/MD.0000000000030273 36221357 10.1097/MD.0000000000030273 PMC9542566

[18] Chen Q, He L, Li Y et al (2022) Risk factors on the incidence and prognostic effects of colorectal cancer with brain metastasis: a SEER-based study. Front Oncol. 10.3389/fonc.2022.758681 35372090 10.3389/fonc.2022.758681 PMC8971714

[19] Mongan JP, Fadul CE, Cole BF et al (2009) Brain metastasis from colorectal cancer: risk factors, incidence, and the possible role of chemokines. Clin Colorectal Cancer. 10.3816/CCC.2009.n.016 19423503 10.3816/CCC.2009.n.016

[20] Zang YW, Gu XD, Xiang JB, Chen ZY (2012) Brain metastases from colorectal cancer: microenvironment and molecular mechanisms. Int J Mol Sci. 10.3390/ijms131215784 23443093 10.3390/ijms131215784 PMC3546661

[21] Salhia B, Kiefer J, Ross JTD et al (2014) Integrated genomic and epigenomic analysis of breast cancer brain metastasis. PLoS ONE. 10.1371/journal.pone.0085448 24489661 10.1371/journal.pone.0085448 PMC3906004

[22] Shim JK, Lim SH, Jeong JH et al (2022) A lignan from *Alnus japonica* inhibits glioblastoma tumorspheres by suppression of FOXM1. Sci Rep. 10.1038/s41598-022-18185-w 35978012 10.1038/s41598-022-18185-w PMC9385634

[23] Zhang K, Zhu L, Fan M. Oxygen, a Key Factor Regulating Cell Behavior during Neurogenesis and Cerebral Diseases. *Front Mol Neurosci*. 2011;4. 10.3389/fnmol.2011.00005 10.3389/fnmol.2011.00005 PMC3073059 21503147

[24] Xia LM, Huang WJ, Wang B et al (2009) Transcriptional up-regulation of FoxM1 in response to hypoxia is mediated by HIF-1. J Cell Biochem. 10.1002/jcb.21996 19097132 10.1002/jcb.21996

[25] Cui J, Xia T, Xie D et al (2016) HGF/Met and FOXM1 form a positive feedback loop and render pancreatic cancer cells resistance to Met inhibition and aggressive phenotypes. Oncogene. 10.1038/onc.2016.14 26876216 10.1038/onc.2016.14 PMC4985506

[26] Chen S, Zhou Y, Chen Y, Gu J (2018) Fastp: an ultra-fast all-in-one FASTQ preprocessor. Bioinformatics. 10.1093/bioinformatics/bty560 30423086 10.1093/bioinformatics/bty560 PMC6129281

[27] Dobin A, Davis CA, Schlesinger F et al (2013) STAR: ultrafast universal RNA-seq aligner. Bioinformatics. 10.1093/bioinformatics/bts635 23104886 10.1093/bioinformatics/bts635 PMC3530905

[28] Patro R, Duggal G, Love MI, Irizarry RA, Kingsford C (2017) Salmon provides fast and bias-aware quantification of transcript expression. Nat Methods. 10.1038/nmeth.4197 28263959 10.1038/nmeth.4197 PMC5600148

[29] Ewels P, Magnusson M, Lundin S, Käller M (2016) MultiQC: summarize analysis results for multiple tools and samples in a single report. Bioinformatics. 10.1093/bioinformatics/btw354 27312411 10.1093/bioinformatics/btw354 PMC5039924

[30] Love MI, Huber W, Anders S (2014) Moderated estimation of fold change and dispersion for RNA-seq data with DESeq2. Genome Biol. 10.1186/s13059-014-0550-8 25516281 10.1186/s13059-014-0550-8 PMC4302049

[31] Yu G, Wang LG, Han Y, He QY (2012) ClusterProfiler: an R package for comparing biological themes among gene clusters. OMICS J Integr Biol. 10.1089/omi.2011.0118 10.1089/omi.2011.0118 PMC3339379 22455463

[32] Fedchenko N, Reifenrath J (2014) Different approaches for interpretation and reporting of immunohistochemistry analysis results in the bone tissue–a review. Diagn Pathol 9:1–12 25432701 10.1186/s13000-014-0221-9 PMC4260254

