# Supplementary material for: FOXM1 expression is induced by the brain microenvironment and supports CRC brain metastatic adaptation
Source: Clin Exp Metastasis. 2026 Apr 27;43(3):22. doi: 10.1007/s10585-026-10397-y (PMC13111526; doi:10.1007/s10585-026-10397-y)
Supplement: Supplementary file 1 — Supplementary file1 (PDF 7863 kb) [file 10585_2026_10397_MOESM1_ESM.pdf]

Supplementary Table S1: **RNA-seq clinical data of CRC BMs and LMs specimens**

Metastatic site	Sex	Age	Tumor cell (%)	Mutation type
Brain	Female	83	80	KRAS
Brain	Female	74	100	KRAS
Brain	Female	72	90	KRAS
Brain	Male	53	80	KRAS
Brain	Male	NA	80	KRAS
Liver	Male	62	80	KRAS
Liver	Female	88	80	KRAS
Liver	Female	70	80	KRAS
Liver	Male	68	80	KRAS
Liver	Female	88	90	KRAS
Liver	Male	80	90	KRAS
Liver	Male	59	90	KRAS

Details of patients with KRAS-mutant metastatic colorectal cancer (CRC): The table summarizes sex, age, tumor cell percent (%), and metastatic site for 12 patients. Metastatic sites include brain and liver. Tumor cell percentages represent the estimated proportion of tumor cells in the analyzed tissue. One patient's age was not available (NA).

Fig. S1: **RNAseq Quality Control and mapping efficiency**

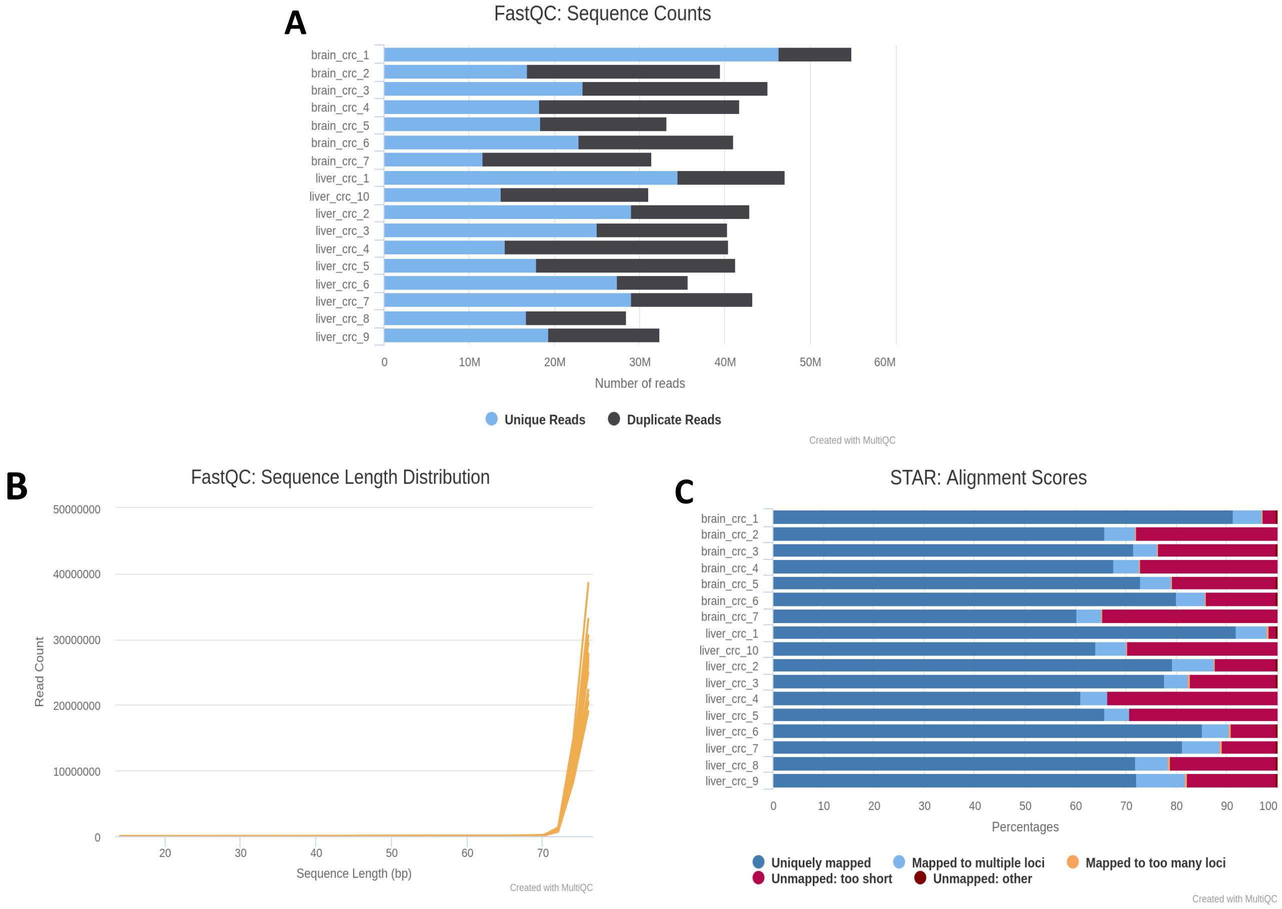

A Total read counts and the proportion of duplicate reads across all samples. Blue bars indicate unique reads, while black bars represent duplicate reads. **B** Sequence length distribution of RNAseq reads, displaying the range and uniformity of read lengths across samples. **C** STAR Alignment Scores, including the proportion of uniquely mapped reads, multi-mapping reads, and unmapped reads across samples.

Supplementary Table S2: **Clinical data of CRC BMs and LMs specimens for IHC staining**

		LM (n=21)	BM (n=21)	P value
Median age (range)		66 (34,85)	67 (34, 85)	0.8904
Sex - no (%)				>0.9999
	Male	13 (62)	10 (48)	
	Female	8 (38)	11 (52)	
Primary- no (%)				>0.9999
	Right	4 (19)	2 (10)	
	Left	7 (33)	4 (19)	
	Rectum	6 (29)	2 (10)	
	NA	4 (19)	13 (61)	
Brain lesion location (Left, Right, NA)				
	Cerebellum		3 (2,0,1)	
	Frontal		2 (1,0,1)	
	Parietal		1 (1,0,0)	
	Occipital		0	
	Temporal		1 (0,1,0)	
	NA		14	
Number of BM				
	1		14 (66)	
	2		2 (10)	
	3		2 (10)	
	4		0	
	5		3 (14)	
BM Symptomatic			21 (100)	

Comparison of patients' characteristics between liver metastasis (LMs) and brain metastasis (BMs) groups in colorectal cancer. The table summarizes key clinical information for patients with LMs (n=21) and BMs (n=21). It includes median age, sex distribution, location of the primary tumor, brain lesion locations (left, right, or not available), number of brain metastasis per patient, and whether the brain metastasis were symptomatic. No significant differences were observed between groups for age, sex, or primary tumor site.

Supplementary Fig. S2: **IHC staining of 18 CRC BMs specimens and 18 CRC LMs specimens**

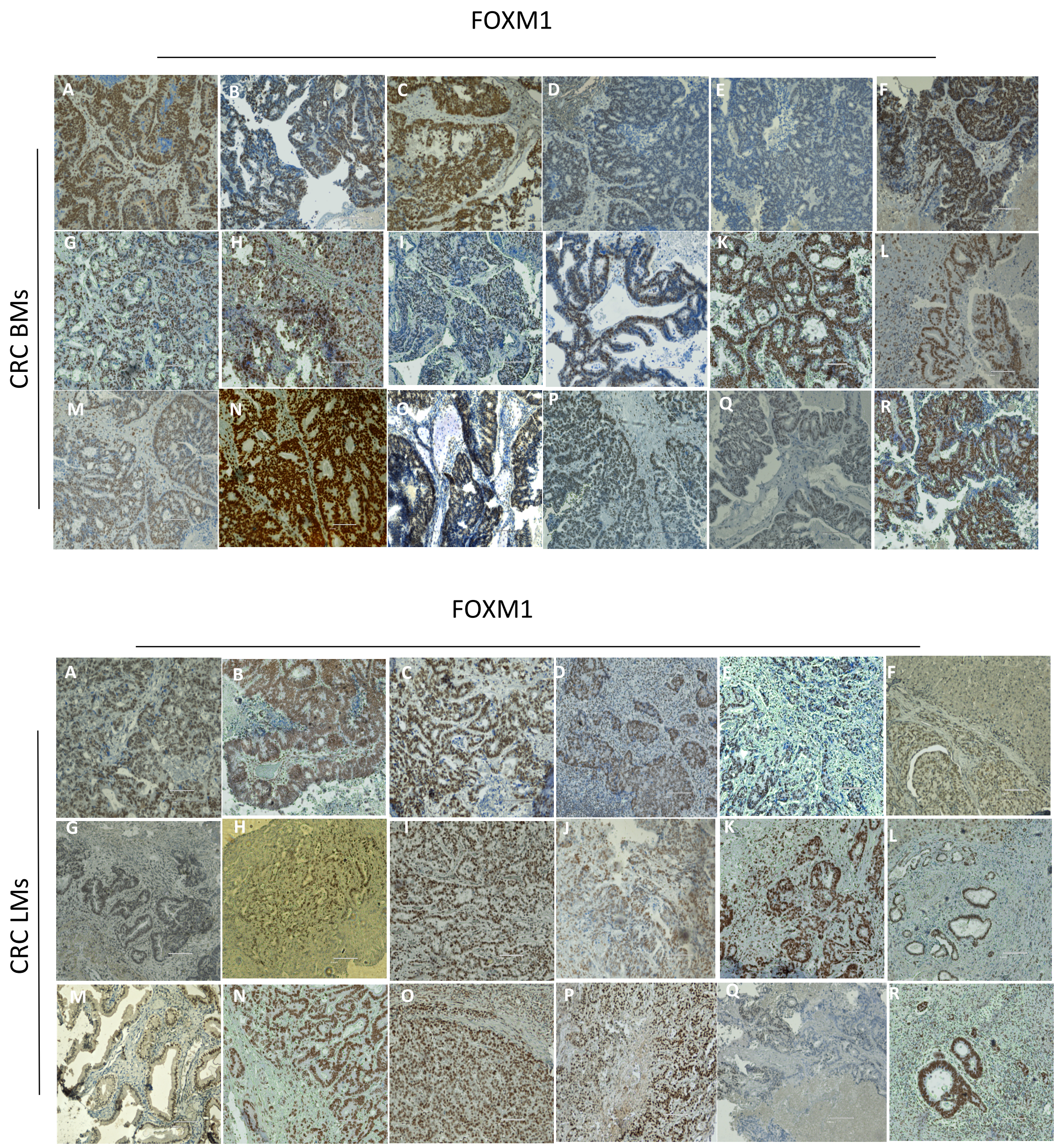

FOXM1 immunohistochemistry (IHC) staining of KRAS-mutant colorectal cancer brain (top panel) and liver metastasis (bottom panel), each 18 samples. Each image corresponds to a different patient. Scale bars = 0.4 mm.
